# Cerebral blood flow in adolescents with drug-naive, first-episode major depressive disorder: An arterial spin labeling study based on voxel-level whole-brain analysis

**DOI:** 10.3389/fnins.2022.966087

**Published:** 2022-07-27

**Authors:** Ying Xiong, Rong-Sheng Chen, Xing-Yu Wang, Xiao Li, Lin-Qi Dai, Ren-Qiang Yu

**Affiliations:** ^1^Department of Radiology, The First Affiliated Hospital of Chongqing Medical University, Chongqing, China; ^2^Department of Hematology, Chongqing General Hospital, Chongqing, China; ^3^Department of Psychiatry, The First Affiliated Hospital of Chongqing Medical University, Chongqing, China

**Keywords:** major depressive disorder, adolescent, arterial spin labeling, regional cerebral blood flow, fMRI

## Abstract

**Purpose:**

The major depressive disorder (MDD) can be a threat to the health of people all over the world. Although governments have developed and implemented evidence-based interventions and prevention programs to prevent MDD and maintain mental health in adolescents, the number of adolescents with this condition has been on the rise for the past 10 years.

**Methods:**

A total of 60 adolescents were recruited, including 32 drug-naive adolescents with first-episode MDD and 28 healthy controls (HCs). Alterations in the intrinsic cerebral activity of the adolescents with MDD were explored using arterial spin labeling (ASL) while differences in the regional cerebral blood flow (rCBF) of the two groups were assessed based on voxel-based whole-brain analysis. Finally, correlations between the regional functional abnormalities and clinical variables were investigated for adolescents with MDD.

**Results:**

Compared with HCs, MDD patients had a lower rCBF in the left triangular part of the inferior frontal gyrus (IFGtriang) but a higher one in the right Precental gyrus (PreCG). Negative correlations were also noted between the CBF in the left IFGtriang and the Hamilton depression scale (HAMD) scores of MDD patients.

**Conclusion:**

Elucidating the neurobiological features of adolescent patients with MDD is important to adequately develop methods that can assist in early diagnosis, precaution and intervention.

## Introduction

Major depressive disorder (MDD), a mental illness characterized by persistent low mood, is linked to significant morbidity and mortality. According to the World Health Organization (WHO), around 350 million people from around the world suffer from depression which largely contributes to illness and disability (Frankish et al., 2018). At the same time, for half of the people diagnosed with mental disorders, the first symptoms generally appear before the age of 14 (Kessler et al., 2007). The case for MDD is not different as its incidence largely increases after adolescence (Kessler et al., 2003). Indeed, while 10–20% of adolescents are estimated to have a mental health disorder (Olfson et al., 2014), the lifetime prevalence of MDD in adolescents exceeds 15% (Kessler et al., 2007). Furthermore, numerous studies have shown some degree of continuity between MDD in adolescents and that of adults (Kessler et al., 2007) since the risk of depression persisting into adulthood is two to four times higher (Pine et al., 1999). Although governments have developed and implemented evidence-based interventions and prevention programs to prevent MDD and maintain mental health in adolescents, between 34 and 75% of them experience a relapse within the first five years since the first depressive events (Kennard et al., 2006). Unfortunately, these cases tend to remain underdiagnosed and undertreated (Olfson et al., 2014).

Early (<30 years) depression and higher heritability (Lyons et al., 1998) as well as suicidal ideation associated with longer symptom duration (Korten et al., 2012) are strongly associated with later relapse (Birmaher et al., 2004).

At present, the pathogenesis of MDD remains unclear, with its clinical diagnosis being mainly based on symptoms (Gao et al., 2019; Yu et al., 2021). At the same time, due to self-reports and doctor bias, misdiagnosis and missed diagnosis may also occur. Nevertheless, with the brain being in a developing state during adolescence (Dennis et al., 2013; Luna et al., 2015), plasticity provides a means for applying additional diagnostic interventions. However, to date, the understanding of the neural basis of adolescent MDD remains limited.

Considering the breadth and severity of MDD in adolescents, in-depth research on its neural mechanism is crucial in order to provide evidence for the early diagnosis of depression, the development of interventions as well as the improvement of current treatments.

Functional neuroimaging provides a means for undertaking neurobiological studies of MDD. Early studies of brain functions in depressed patients mainly relied on the positron emission tomography (PET) and the single photon emission computed tomography (SPECT) to measure glucose metabolism and the baseline regional cerebral blood flow (rCBF) (Seminowicz et al., 2004; Smith and Cavanagh, 2005). However, both approaches are not free from significant disadvantages which include the use of radioactive materials, the risk of allergy, invasiveness, poor spatial resolution, limited scope of use, high price, and risk factors that limit their use in adolescents with depression (Ho et al., 2013; Chen et al., 2016). In contrast, blood oxygenation level dependence (BOLD) is not only non-radioactive but it is also easily accessible as well as reliable (Fox and Raichle, 2007; Greicius, 2008; Detre et al., 2009). However, since the measurement of BOLD signals relies on vascular factors and is sensitive to blood flow and blood volume, results may still be influenced by confounding factors (Wintermark et al., 2005). Furthermore, BOLD indirectly measures neuronal activation without focusing on cerebral perfusion and hence, measures of neural activity tend to be relative instead of absolute. As such, they cannot be well applied to the study of the neurophysiology of depression.

Emerging arterial spin labeling (ASL) perfusion magnetic resonance imaging (MRI) is a technique that applies magnetically labeled arterial blood water as an endogenous tracer to quantitatively assess rCBF (Ma et al., 2017; Zheng et al., 2019; Momosaka et al., 2020; Wang et al., 2022). This approach not only provides results which are consistent with PET and SPECT (Chen et al., 2011; Boscolo Galazzo et al., 2016; Tosun et al., 2016; Verclytte et al., 2016), but it also enables absolute quantification of rCBF (Detre et al., 2009; Bokkers et al., 2010), thus presenting ASL as a reliable physiological marker of neural activity (Akgoren et al., 1994; Hoge and Pike, 2001; Lauritzen, 2001). Assessing whether the rCBF pattern in high-risk groups for depression deviates significantly from the norm may be helpful for early diagnosis, intervention and prevention (Bonne and Krausz, 1997). In addition, in various neuropsychiatric disorders, perfusion patterns can act as objective biomarkers which help to track disease progression as well as developmental brain maturation (Brown et al., 2007; Borogovac and Asllani, 2012). In these contexts, ASL offers several advantages in terms of its safety, non-invasiveness, non-radioactivity, ease to obtain, reproducibility, its ability to quantitatively display perfusion abnormalities, its high spatial resolution and its good accuracy. Altogether, these features make ASL a powerful tool for the study of rCBF in patients with depression (Wang et al., 2011; Ho et al., 2013). In particular, it has the potential to be applied in the study of the neural basis and for the early diagnosis of depression.

Previous studies have mostly recruited patients with long-term diseases and those undergoing drug treatment (Duhameau et al., 2010; Vasic et al., 2015) as different antidepressants may have potentiating or weakening effects on rCBF (Nobler et al., 2002). This fact causes the research to be concentrated on adult patients (Vasic et al., 2015; Fu et al., 2017), thereby making it unsuitable for considering the early diagnosis and treatment of adolescent depression. Therefore, this study, involving first-episode untreated adolescents with major depressive disorder, may help in reducing confounding factors, in resolving inconsistencies in the existing literature, and eventually in determining whether hyperactive perfusion or hypoperfusion is indeed responsible for adolescent MDD potential biomarkers.

Consequently, ASL was applied for testing the following hypotheses: (1) the rCBF values of the relevant brain regions in patients with MDD can change compared to the control group and (2) the changes in rCBF values are linked to the severity of the disease.

## Materials and methods

### Participant selection

This study recruited 40 adolescents between 12 and 17 years old and diagnosed with MDD from the inpatient clinics of the First Affiliated Hospital of Chongqing Medical University, China. Two experienced psychiatrists independently evaluated the presence or absence of diagnoses based on the Mini International Neuropsychiatric Interview for Children and Adolescents (MINI-KID). The validated and reliable Chinese version of the 17-item depression scale Hamilton depression scale-17 (HAMD-17) was then used to assess the severity of depression. The HAMD-17 instrument was selected on account of its extensive application in clinical practice and scientific research. On the day of MRI-based assessments, the patients obtained a total score of at least 17 on the HAMD-17 scale.

The exclusion criteria for the participants were as follows: being left-handed; drug misuse; intense physical diseases or any other somatic ones; a history of neurological disorders running in the family; head motion exceeding 3 mm in translation or 3° in rotation; the presence of any surgically-placed electronic or metal materials that could interfere with functional MRI (fMRI) assessment; the existence of other psychiatric disorders such as personality disorders or schizophrenia; the presence of abnormal cerebral structures after early MRI scanning. In the case of the healthy controls (HCs), the inclusion criteria were: aged 12–17 years; being from the Han Chinese population; no previous history of psychiatric diagnosis; no history of severe neurological (e.g., stroke, concussion) or medical conditions (e.g., chronic inflammatory or autoimmune diseases); no conditions affecting metabolism (e.g., hypertension, diabetes, or thyroid dysfunction). The same set of exclusion criteria was applied to both groups. Overall, 32 patients as well as 28 healthy participants were controlled for variations in factors such as educational status, age and gender, with the same exclusion standards as depressive patients.

Prior to participation in the current study, a written informed consent was signed by all participants. The study was also performed with the approval of the Medical Ethics Committee of the First Affiliated Hospital of Chongqing Medical University in accordance with the Helsinki’s Declaration.

### Image acquisition

Magnetic resonance images were acquired with a 3.0 Tesla GE Signa HDx system (General Electric Healthcare), housed in the department of Radiology of the First Affiliated Hospital of Chongqing Medical University.

After lying down for the scans, participants were required to remain quiet, close their eyes without thinking of anything in particular and to relax without moving. Throughout the scanning process, they were required to remain awake. Their hearing was also protected with earplugs while head movement was minimized with the help of a sponge. A standard eight-channel head coil was used with the MRI scanner. Structural abnormalities as well as intracranial lesions were excluded when acquiring 3D T1WI, T2WI, and T2 FLAIR sequences and the resting state perfusion imaging was performed using a 3D ASL with the following parameters: NEX 3, TE 9.8 ms, TR 4,639 ms, FOV 240 mm × 240 mm, slice thickness 4.0 mm, post label delay time 1,525 ms, 3D spiral k-space filling, points 512, arms 8, acquisition scan slices 40, acquisition time 4 min 29 s.

### Image processing

Quality control of results was performed by one experienced radiologist who directly checked the data acquired on the MR scanner. In this case, the scanning process was repeated when motion artifacts were observed as a result of head movements. The dcm2nii software^1^ was then used to convert digital imaging and communications in medicine (DICOM) images of CBF into neuroimaging informatics technology initiative (NIFTI) formats before running SPM12^2^ in MATLAB 2013b. To normalize images, those of each participant were registered for a one-step registration method before subsequently checking the image quality. This was followed by image standardization and spatial smoothing with dpabi4.3^3^ and SPM12, respectively. The full width at half-maximum (FWHM) of the Gaussian kernel was 8 mm with the removal of non-brain tissues.

### Statistical analyses

All data were analyzed using SPSS22 (Chicago, IL, United States) software. Education level, gender, age, and rCBF were compared between groups using two-sample *t*-tests, with regression analysis also performed using years of education, gender and age as covariates. In this case, a False Discovery Rate (FDR) correction of 0.050, or an uncorrected *p*-value at 0.001 was selected as the threshold for statistical significance. The above steps were completed with SPM12. The CBF values of brain regions showing differences were extracted with dpabi4.3 before visualizing results in Xjview10. The relationship between rCBF values and HAMD scores were eventually determined based on Spearman correlational analysis, with *p*-values of <0.05 considered to be statistically significant.

## Results

### Demographic and clinical data

Table 1 summarizes the demographic characteristics of HCs and MDD patients. The MDD patients and the healthy control groups were not significantly different from each other in terms of education level, gender and age (*p* > 0.05).

**TABLE 1:** Demographic characteristics of healthy controls (HCs) and major depressive disorder (MDD) patients.

Demographic data	HCs (*n* = 28)	MDD (*n* = 32)	*T* (orx^2^)	*p*-value
Gender (male/female)	28(4/24)	32(4/28)	0.010	0.922^a^
Age (years)	15.07 ± 3.90	14.31 ± 1.31	1.592	0.117*^b^*
Years of education (years)	9.75 ± 2.38	9.06 ± 1.63	1.320	0.192^b^
HAMD score	0.18 ± 0.48	25.22 ± 5.16		

HAMD, Hamilton depression scale. ^a^The p-value for gender distribution was obtained by chi-square test. ^b^The p-value were obtained by two sample t-tests.

### Differences in regional cerebral blood flow between healthy controls and major depressive disorder patients

**FIGURE 1:**
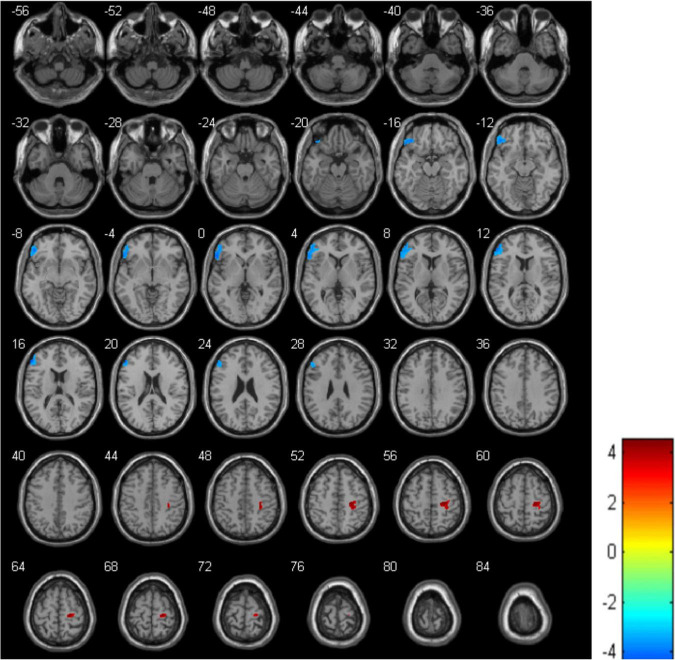
Differences in regional cerebral blood flow (rCBF) between healthy controls (HCs) and major depressive disorder (MDD) patients. The blue area represents a decrease in rCBF while the red one represents an increase in rCBF.

The analysis showed that, compared with the HCs, the MDD group displayed a decrease in rCBF in the left IFGtriang (cluster = 1178, and peak *t* = 4.2955), along with an increase in the right PreCG (cluster = 384, and peak *t* = −4.5095) (Figure 1 and Table 2). In this case, a *p*-value of <0.001 was selected as the cluster threshold.

**TABLE 2:** Significant differences in regional cerebral blood flow (rCBF) values between healthy controls (HCs) and major depressive disorder (MDD) patients.

Cluster location	Peak (MNI)			Number of voxels	*T*-value
	
	X	Y	Z		
Left IFGtriang	−54	40	16	1178	4.2955
Right PreCG	22	−24	60	384	−4.5095

MNI, Montreal Neurological Institute; IFGtriang, triangular part of the inferior frontal gyrus; PreCG, Precental gyrus.

### Correlation between regional cerebral blood flow and Hamilton depression scale scores

**FIGURE 2:**
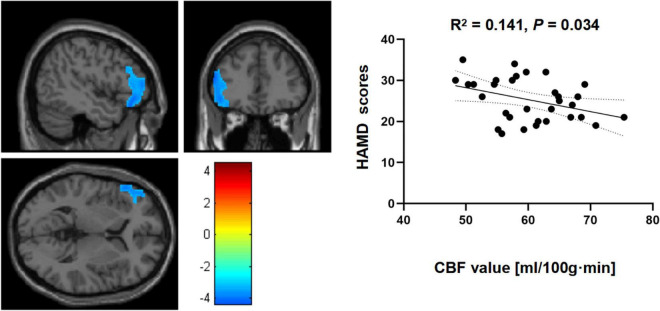
Correlational analyses between cerebral blood flow (CBF) in the left triangular part of the inferior frontal gyrus (IFGtriang) and Hamilton depression scale (HAMD) scores for the two groups.

Correlational analysis indicated a negative but significant relationship between HAMD scores and rCBF changes in the left IFGtriang when HCs and MDD patients were compared (*R*^2^ = 0.141, *p* = 0.034) (Figure 2). However, for the right PreCG, similar comparisons between the two groups did not yield significant correlations between Hamilton depression scale (HAMD) scores and rCBF changes (*R*^2^ = 0.038, *p* = 0.286).

## Discussion

Voxel-based whole-brain analysis was performed based on arterial spin labeling imaging for calculating rCBF (Wang et al., 2022). Compared with the traditional fMRI data analysis, ASL provides quantitative and reproducible whole-brain measures that are closely related to neural activity for local cerebral blood flow. It also has low inter-subject variability while providing quantitative measures of cerebral blood flow which directly reflect neural activities as well as brain physiology (Haller et al., 2016).

The current study used ASL techniques for quantitatively investigating rCBF in patients diagnosed with MDD. The results confirmed the hypotheses that the rCBF values of the relevant brain regions in MDD patients could change compared to the control group, with this observation corroborating previously-reported findings (Cooper et al., 2020). This analysis revealed reduced rCBF in the right parahippocampus, thalamus, fusiform, and middle temporal gyrus as well as the left and right insula, for those with MDD. After comparing the rCBF in the current study, two abnormal brain regions, namely the left IFGtriang and the right PreCG were identified. Furthermore, subsequent correlational analyses indicated that depression severity (HAMD scores) in MDD was significantly correlated to altered CBF in the left IFGtriang. Overall, the results suggest that altered CBF in the left IFGtriang could represent a potential neural biomarker of vulnerability to MDD.

The influence of rCBF on the pathogenesis of MDD remains unclear but previous studies have shown that different genotypes could have some effects on cerebral blood flow metabolism during depression (Rao et al., 2007; Li et al., 2016). In recent years, numerous studies (Crookes et al., 2018; Nanayakkara et al., 2018) have consistently suggested that high-risk factors for cerebrovascular diseases such as hypertension, diabetes, and atherosclerosis could also influence the occurrence of depression. It was further found that changes in CBF could be the result of changes in neurotransmitters such as dopamine, serotonin, catecholamines, and glutamate as well as gamma-aminobutyric acid (GABA) that play a role in regulating vascular responses (Dukart et al., 2018). Correlations between cerebral blood flow and local neural activity and metabolism are known as neurovascular coupling (Zhu et al., 2017; Chen et al., 2022). This feature depends on the integrity of the neurovascular unit as altered neuronal activity can lead to changes in blood flow.

Studies of the frontal lobe, involved in higher-order cognitive control and emotional processing, have, so far, yielded the most consistent findings in MDD (Zhang et al., 2018; Zhao et al., 2021). In particular, as an important part of the frontal lobe, the left IFGtriang plays a vital role in anhedonia and emotional activities (Hou et al., 2021) in patients with MDD as it is involved in controlling attention and regulating emotions.

The results finally showed that abnormal rCBF values were negatively correlated to the left IFGtriang and HAMD scores, and this could possibly be attributed to rumination and emotional processing in depressive patients.

## Limitations

In addition to its small sample size, this study could not totally exclude physiological noises such as heart and respiratory rhythms in the resting state. Secondly, the age of the patients varied between 12 and 17 years and possible confounding variables regarding age differences could not be removed. Thirdly, the HAMD-17 is yet to be fully validated for rating the severity of depression in adolescent samples. Finally, some patients initially considered to have uni-polar depressions were subsequently diagnosed with bipolar ones in clinical practice.

## Conclusion

This study, in comparing HCs and MDD patients, highlighted discrepancies in their corresponding rCBF values. In particular, compared with healthy participants, drug-naive adolescents with a first-episode of MDD had lower rCBF in the left IFGtriang and the right PreCG. Finally, abnormal rCBF values in the left IFGtriang were found to be negatively correlated with HAMD scores, hence suggesting that the left IFGtriang could be an area of interest for better understanding the neurobiology of MDD.

## Data availability statement

The raw data supporting the conclusions of this article will be made available by the authors, without undue reservation.

## Ethics statement

The studies involving human participants were reviewed and approved by the Medical Ethics Committee of the First Affiliated Hospital of Chongqing Medical University. Written informed consent to participate in this study was provided by the participants’ legal guardian/next of kin. Written informed consent was obtained from the individual(s), and minor(s)’ legal guardian/next of kin, for the publication of any potentially identifiable images or data included in this article.

## Author contributions

YX: writing—original draft. R-SC: writing—original draft and data analyses. X-YW: scanning MRI. XL and L-QD: investigation. R-QY: conceptualization, checking the data, methodology, writing—review and editing, resources, supervision, project administration, and final approval. All authors contributed to the article and approved the submitted version.

## Footnotes

1
https://neuroelf.net/wiki/doku.php?id=dcm2nii

2
https://www.fil.ion.ucl.ac.uk/spm/software/spm12/

3
http://rfmri.org/dpabi
